# An Incessant Tachycardia with Alternating QRS Complexes: What Is the Mechanism?

**DOI:** 10.19102/icrm.2022.130203

**Published:** 2022-02-15

**Authors:** Mustafa Cetin, Ender Ornek, Serdal Bastug, Meryem Kara, Bulent Deveci, Ahmet Korkmaz, Ozcan Ozeke, Serkan Cay, Firat Ozcan, Serkan Topaloglu, Dursun Aras

**Affiliations:** ^1^Department of Cardiology, Health Sciences University, Ankara City Hospital, Ankara, Turkey; ^2^Department of Cardiology, Ankara Yıldırım Beyazıt University School of Medicine, Ankara City Hospital, Ankara, Turkey

**Keywords:** Alternating beats, double exit, fascicular VT, His–Purkinje system, HPS, multiple exits, wide QRS tachycardia

## Abstract

We present a patient with ischemic cardiomyopathy who had ventricular tachycardia (VT) with QRS morphology alternans. The electrophysiological findings, in this case, supported the occurrence of antegrade activation of the proximal His–Purkinje system during VT, with the ultimate electrocardiogram morphology dependent on fusion from intramyocardial and His–Purkinje activations.

## Case presentation

**Figure 1:**
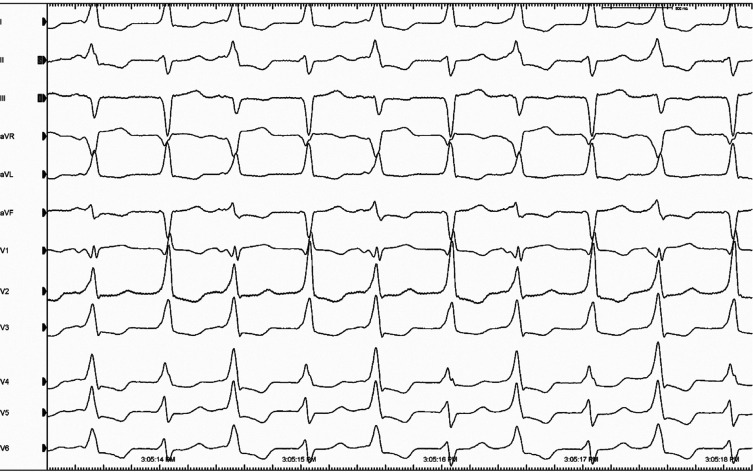
Electrocardiogram showing a beat-to-beat shift in the QRS axis and amplitude occurring in a 1:1 fashion without change in the mean cycle length.

**Figure 2:**
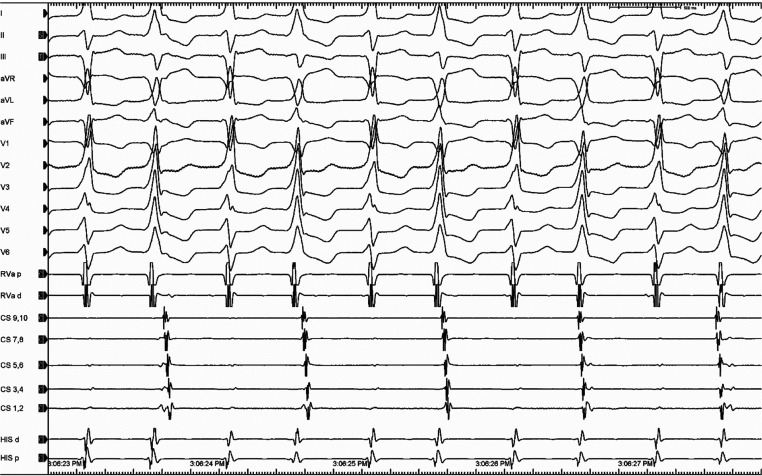
Intracardiac recording during tachycardia shows an atrioventricular (AV) dissociation confirming the diagnosis of VT. *Abbreviations*: CS, coronary sinus; d, distal; p, proximal; RVa, right ventricular apex.

A 74-year-old woman with a history of inferoposterolateral wall myocardial infarction (MI) and coronary artery bypass grafting presented with incessant tachycardia. A cardiac ultrasonographic study showed an ejection fraction of 30% and moderate-to-severe mitral regurgitation. Electrocardiography revealed a beat-to-beat shift in both the QRS axis and amplitude occurring in a 1:1 fashion without change in a mean cycle length (CL) of 500 ms **(Figure 1)**. Intracardiac recording during tachycardia showed an atrioventricular dissociation during the ventricular tachycardia (VT) **(Figure 2)**. What is the mechanism of the alternating QRS complexes?

## Discussion

Multiple VT morphologies were observed in a majority (60%–80%) of post-MI patients who underwent catheter ablation.^1^ Monomorphic VTs with alternating QRS morphologies have been observed to occur spontaneously and during programmed stimulation in human hearts.^1–7^ Bidirectional VT is one of the most commonly known prototypes of electrical alternans and is characterized by a rapid, wide-complex electrocardiogram (ECG) pattern with alternating QRS morphology and axis.^8,9^ Multiple exit sites from reentrant circuits would be associated with these changing QRS morphologies^2^; however, 3 more options should also be considered: whether more than one arrhythmia is occurring, whether fusion with sinus beats is occurring, or whether tachycardia originates within or in the proximity of the His–Purkinje conduction system (HPS).^10,11^ Identification of the mechanisms for these changes is important when considering the feasibility of ablation as therapy.^1,4^

In addition to a scar-based reentry, the subendocardial Purkinje fibers along the border zone of the subendocardial scar can play an important role in ventricular arrhythmia in post-MI patients.^12^ The phenomenon of an early invasion of the HPS may have an exit point near the preserved conduction system, especially in the septal area.^12–16^ However, the involvement of the HPS in VT associated with heart disease can be difficult to recognize in post-MI patients and has 3 distinct clinical forms as mainly re-entrant or focal mechanisms in distal HPS.^16^ A focal mechanism is present in up to 9% of VTs in patients with prior MI who are induced during the electrophysiology study for radiofrequency ablation.^17^ Furthermore, VT exhibits a focal activation pattern on the endocardial and epicardial surfaces despite a re-entrant VT.^18^

**Figure 3:**
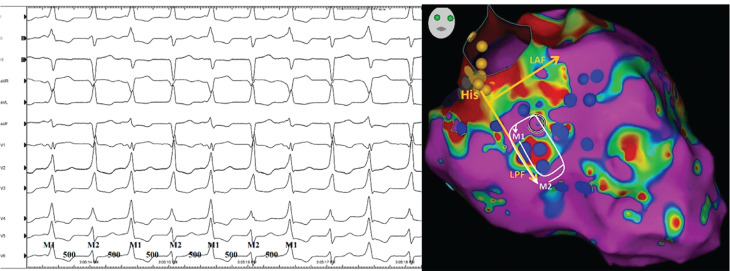
Electrocardiography showing a beat-to-beat alternans in the QRS axis and amplitude occurring in a 1:1 fashion without change in the mean cycle length **(A)** and the possible mechanism of this alternating QRS morphology **(B)**. *Abbreviations*: CL, cycle length; LAF, left anterior fascicle; LPF, left posterior fascicle; M1, morphology 1; M2, morphology 2.

**Figure 4:**
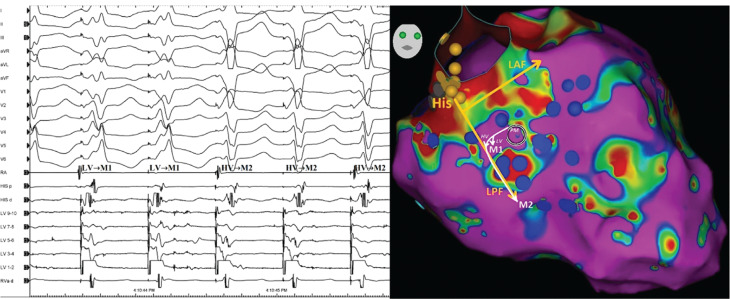
Pace-mapping near the exit of an isthmus during sinus rhythm showing similar morphologies (M1 and M2) of ventricular tachycardias. *Abbreviations*: d, distal; HV-PM, high-voltage pace-mapping; LAF, left anterior fascicle; LPF, left posterior fascicle; LV, left ventricle; LV-PM, low-voltage pace-mapping; M1, morphology 1; M2, morphology 2; p, proximal; PM, pace-map; RA, right atrium; RVa, right ventricular apex.

Differentiating focal VTs from re-entrant VTs is important because the characteristics of the ablation site significantly differ depending on the mechanism.^17^ If the arrhythmia is a VT, then whether the tachycardia is automatic or re-entrant cannot be determined by a 12-lead ECG (nor, for that matter, can it be determined by intracardiac electrograms or activation maps rendered via an automated system).^19,20^ Only pacing maneuvers (specifically, entrainment mapping) can identify the mechanism of arrhythmia. In **Figure 3**, a VT focus that arises within the interventricular septum may sometimes propagate into the HPS and exit into the ventricular myocardium fairly distant from the original focus (posterior fascicule in the current case). This can mistakenly suggest that the true site of origin is at the exit site of the heart’s normal conduction system. Furthermore, the re-entrant monomorphic VT originating from the left posterior Purkinje fibers, which is analogous to an idiopathic left VT, can develop in the acute or chronic phase of MI **(Figure 4)**. Hayashi et al. described 4 patients with prior MI in whom VT involving the HPS was observed.^15^ Penetration of the HPS by myocardial VT, with resultant fusion from intramyocardial VT and HPS activation, could have accounted for the near-normalization of the QRS morphology during VT2.^14^ Bogun et al. described 9 patients with an ischemic VT, a relatively narrow QRS complex (≤145 ms), and HPS involvement but excluded bundle branch re-entry, interfascicular re-entry, and fascicular VT as a mechanism.^12^ In the current case, there was no clear evidence of conducted sinus beats giving rise to capture and fusion; however, the morphology of the QRS complex during VT1 exhibited a more distinctively inferior axis (M1, morphology 1 in **Figure 3**) than that during VT2 (M2, morphology 2 in **Figure 3**) with a similar CL such that one re-entrant loop with varying degrees of fusion is likely **(Figure 3)**.^10^ We performed both high- and low-output pacing to more clearly elucidate the mechanism **(Figure 4)**. When tachycardias originate in the conduction system, the pace-map at the site of origin (within the conduction system) may be completely different from the VT QRS morphology because of concomitant capture of the surrounding myocardium near the conduction tissue, whereas, during tachycardia, the exit is at a distal site into the myocardium. Therefore, both high- and low-output pacing should be considered **(Figure 4)**, especially when the tachycardia shows spontaneous variation in QRS duration or when multiple early sites are noted when mapping with a 3-dimensional system.^10^ On the other side, the pace-mapping of VT circuits may be difficult where there are multiple entrances and exits,^21^ and it should be kept in mind that pace-mapping at the high output may capture the myocardium of a contralateral chamber and result in spurious findings.

**Figure 5:**
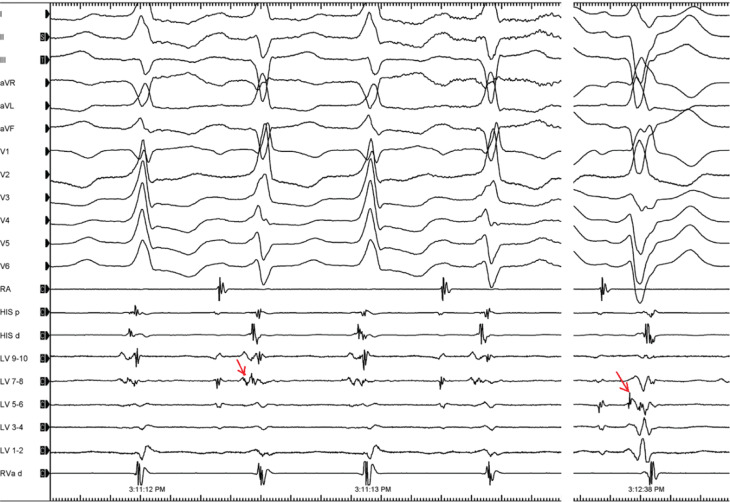
Presystolic Purkinje potentials are seen at successful ablation points. d: distal; LV: left ventricle; p: proximal; RA: right atrium; RVa: right ventricular apex.

As there was no change in the CL despite QRS alternans, we considered it to be a single re-entrant loop with 2:1 penetration of the HPS by myocardial VT, with resultant fusion from intramyocardial VT and HPS activation. The precise electrophysiological factors underlying direct HPS penetration remain speculative. It is difficult to explain why this sudden change in the VT configuration occurred without any specific maneuver. The presence of spontaneous changes in the regional refractoriness of the HPS or the adjacent ventricular myocardium that may result in an antegrade or retrograde block of the HPS during the wide QRS complex VT might have caused these alternans. Another possible explanation could be the presence of 2 very near but distinct exit points of the tachycardia circuit, 1 connecting to the HPS directly and the other 1 connecting to the ventricular myocardium, resulting in a similar QRS configuration but a different width. Catheter ablation was highly effective in eliminating this VT without affecting left ventricular conduction.^15^ Bogun et al. identified a Purkinje potential at each successful ablation site.^12^ Radiofrequency ablation of presystolic Purkinje potentials in the para-Hisian region in the current case **(Figure 5)** resulted in the termination of the VT,^15^ and, from then on, the patient was non-inducible for VTs. Whether uncovering focal and re-entrant VT mechanisms routinely during ablation procedures will improve the outcomes of VT ablation is worthy of future study.^20^
